# A Simple Aptamer SERS Sensor Based on Mesoporous Silica for the Detection of Chlorpyrifos

**DOI:** 10.3390/foods11213331

**Published:** 2022-10-23

**Authors:** Sa Dong, Qiuyun Shi, Kangli He, Jianwei Wu, Zixin Zhu, Jianguo Feng

**Affiliations:** College of Plant Protection, Yangzhou University, Yangzhou 225009, China

**Keywords:** aptamer, mesoporous silica nanoparticles, surface-enhanced Raman spectroscopy, chlorpyrifos, residue

## Abstract

Chlorpyrifos is an organophosphorus insecticide, which can be used to control a variety of chewing and piercing mouthparts pests in agricultural production. It can destroy the normal nerve impulse conduction by inhibiting the activity of acetylcholinesterase or cholinesterase in the nerves, causing a series of poisoning symptoms. In order to achieve the quantitative analysis of chlorpyrifos residues in agricultural products, an aptamer-controlled signal molecule release method was developed in this study. The signal molecule 4-ATP of surface-enhanced Raman spectroscopy (SERS) was loaded into aminated mesoporous silica nanoparticles (MSNs-NH_2_) prepared by the one pot method, and then coated with an aptamer of chlorpyrifos through electrostatic interaction. The specific binding of the aptamer and chlorpyrifos led to the release of 4-ATP, and the amount of 4-ATP released was positively correlated with the amount of chlorpyrifos. Finally, the standard curve of chlorpyrifos quantitative detection based on SERS was established. Meanwhile, Ag-carrying mesoporous silica (Ag@MSNs) was prepared as the reinforcement substrate for SERS detection. The results showed that there was a good linear correlation between the Raman intensity and the concentration of chlorpyrifos at 25–250 ng/mL, and the limit of detection (LOD) was 19.87 ng/mL. The recoveries of chlorpyrifos in the apple and tomato samples were 90.08–102.2%, with RSD < 3.32%. This method has high sensitivity, specificity, reproducibility and stability, and can be used for the quantitative detection of chlorpyrifos in the environment and agricultural products.

## 1. Introduction

Organophosphates are widely used as insecticides and are considered to have strong neurotoxicity because they can irreversibly inhibit the activity of acetylcholinesterase in the human body and cause serious damage to the nervous system [1,2]. Chlorpyrifos is a highly effective, broad-spectrum organophosphorus insecticide, which is commonly used to control a variety of chewing and piercing mouthpart pests on rice, wheat, fruit trees, vegetables, and other crops [3]. The irrational use of chlorpyrifos can lead to its residue in the environment and agricultural products, thus causing great harm to humans. In recent years, the problem of pesticide residues in agricultural products has become the focus of public attention. Therefore, the convenient and rapid detection of chlorpyrifos residues in agricultural products is of great importance.

At present, chlorpyrifos residues are mainly detected by instrumental methods such as gas chromatography (GC) [4,5], gas chromatography-mass spectrometry (GC-MS) [6,7,8,9], high performance liquid chromatography (HPLC) [10], and liquid chromatography-mass spectrometry (LC-MS) [7,11]. Although these detection methods have the advantages of high accuracy and sensitivity, they require expensive instruments, professional technicians, tedious sample pretreatment processes, and large amounts of organic solvents, which limit their use for the rapid detection of chlorpyrifos residues in the field. In addition, the immunosorbent assay has also been applied to the detection of chlorpyrifos, which is simple, convenient, sensitive, and does not require the use of expensive instruments, but the antibody preparation process is complex and the cycle is long [12,13]. Therefore, it is necessary to develop a convenient, rapid, and sensitive method to detect chlorpyrifos residues in the environment and agricultural products.

Aptamers are random sequences with high specificity and affinity for target substances, which are screened from random oligonucleotide libraries in vitro through systematic evolution of ligands by exponential enrichment (SELEX) technology [14,15]. Compared with other recognition molecules, aptamers have the advantages of easy synthesis and modification, high affinity, strong specificity, low cost, good stability, low molecular weight, and repeated denaturation and refolding, which have been widely used in the fields of environmental monitoring and food analysis [16,17].

Surface-enhanced Raman spectroscopy (SERS) technology refers to the process after molecules are adsorbed on the surface of some rough metals (such as gold, silver, and copper), where the electromagnetic field on the metal surface or near surface is enhanced in the excitation area, which enhances the Raman signal intensity of adsorbed molecules [18]. SERS has the advantages of high detection sensitivity, simple sample pretreatment, fast analysis speed, low detection cost, and in situ detection, which has been used for the rapid detection of food additives and pesticide residues in food and agricultural products [19,20]. The SERS enhanced effect was closely related to the substrate material. In general, the precious metals Au, Ag, and Cu had a better SERS enhanced effect, and the surface enhancing factor (SEF) of Ag was the highest (up to 10^6^) [21]. Good SERS substrate should have the characteristics of high SERS activity, high stability and reproducibility, uniform size, and no signal interference from impurity molecules [22,23]. The hot spots generated by a single metallic sol particle are random, because the aggregated nanoparticles are unstable and their deposition under the action of gravity will cause fluctuations in the intensity and number of hot spots during detection. Researchers have usually added chemicals and inert materials to modify the surface of metallic sol particles to prepare a highly active SERS composite substrate to ensure the good reproducibility of the SERS signal [24]. Silica is a representative inert carrier with no absorbance in the visible wavelength range, making it a satisfactory material for shell preparation. At present, mesoporous silica nanoparticles (MSNs) have been widely reported to be used in catalysis, adsorption, sensing, and sustained release due to a series of advantages such as a large surface area and pore volume, tunable morphology and structure, and good biocompatibility, showing great potential in the field of agriculture [25,26,27].

**Figure 1:**
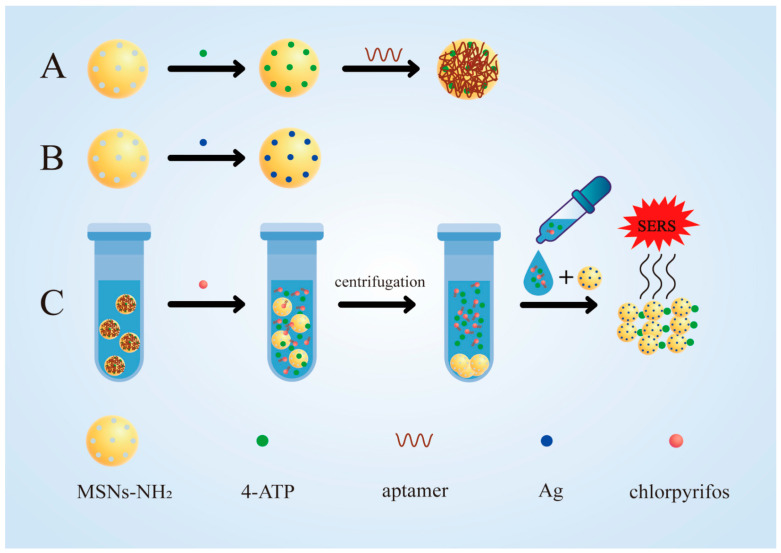
The synthesis of 4-ATP@MSNs-NH_2_@aptamers (**A**) and the Raman substrate Ag@MSNs (**B**); the detection process for chlorpyrifos (**C**).

In this study, a simple and sensitive aptamer sensor for chlorpyrifos detection based on MSNs was prepared. First, aminated mesoporous silica nanoparticles (MSNs-NH_2_) were prepared and loaded with signaling molecules (4-ATP) to produce 4-ATP@MSNs-NH_2_ (Figure 1A). Then, aptamers were adsorbed on 4-ATP@MSNs-NH_2_ through electrostatic interaction, which play the role of gating and control the release of 4-ATP (Figure 1A). At the same time, Ag-carrying MSNs (Ag@MSNs) were prepared as the Raman substrate, which have high activity, good stability, uniform size, and can amplify the Raman signal well (Figure 1B). In the presence of chlorpyrifos, the specific binding of the aptamer to chlorpyrifos results in the release of 4-ATP, which is subsequently enhanced by the Raman substrate Ag@MSNs and detected by Raman spectroscopy (Figure 1C). Finally, an aptamer-gated chlorpyrifos detection method based on the release of signal molecules in MSNs was developed. Compared with other SERS sensors, the detection process of this SERS sensor was simple and fast, requiring only one step of centrifugation to separate the released signal molecules from MSN to conduct SERS detection, without using other instruments, and the whole detection process took a short time (about 40 min) [28,29]. This method can realize the rapid quantitative detection of chlorpyrifos residues in the environment and agricultural products, and provides a novel strategy for the application of SERS technology in immunoassays.

## 2. Experimental Method

### 2.1. Materials and Instruments

Apples (Red Fuji) and tomatoes (Provence) that were chlorpyrifos free identified by HPLC-MS/MS were commercially available. Cetyl trimethyl ammonium bromide (CTAB, 98%), tetraethoxysilane (TEOS, 99.9%), 3-aminopropyl trethoxy silane (APTES, 98%), 3-mercaptopropyl trimethoxy silane (MPTMS, 95%), and rhodamine-6G (R6G) were obtained from Adamas Reagent Co. Ltd. (Shanghai, China). 4-ATP (97%) was provided by Shanghai Energy and Chemical Co. Ltd. (Shanghai, China). Silver nitrate (AgNO_3_), ammonia, methanol, ethanol (99.9%), and hydrochloric acid (6% HCl) were supplied by Sinopharm Chemical Reagent Co. Ltd. (Shanghai, China). Chlorpyrifos (97%), acetamidine (99%), cyhalothrin (98%), and carbendazim (98%) were supplied by Shanxi Qixing Pesticide Co. Ltd. (Shanxi, China). The aptamer DNA fragment of chlorpyrifos was synthesized by Sangon Biotech Co. Ltd. (Shanghai, China). The DNA sequence was as follows: 5′-CCT GCC ACG CTC CGC AAG CTT AGG GTT ACG CCT GCA GCG ATT CTT GAT CGC GCT GCT GGT AAT CCT TCT TTA AGC TTG GCA CCC GCA TCG T-3′.

Transmission electron microscopy (TEM) was undertaken by Tecnai G2 F30 S-Twin and purchased from FEI Co. Ltd. (Hillsboro, OR, USA). X-ray diffraction (XRD) spectra were obtained on SmartLab SE and purchased from Rigaku Corporation (Tokyo, Japan). Fourier transform infrared spectroscopy (FTIR) was obtained by Antains II and purchased from Thermo Fisher Scientific (Waltham, MA, USA). X-ray photoelectron spectroscopy (XPS) data were supplied by EscaLab 250Xi (Thermo, Waltham, MA, USA). All ultraviolet–visible (UV–Vis) absorption spectrum data were obtained with an L5S spectrophotometer and purchased from INESA Co. Ltd. (Shanghai, China). The zeta potentials were recorded using a ZS90 Nano instrument and purchased from Malvern Co. Ltd. (Malvern, UK). The SERS spectra were obtained on an InVia Raman spectrometer and purchased from Renishaw (London, UK).

### 2.2. Synthesis of MSNs-NH_2_ and 4-ATP@MSNs-NH_2_

First, MSNs-NH_2_ was prepared as follows: the aqueous phase was prepared by dissolving 0.1 g CTAB in 70 mL deionized water, and the oil phase was prepared by mixing 30 mL ethanol with 1 mL TEOS. The above two solutions were mixed, and 0.7 mL APTES was slowly dropped in, followed by 0.5 mL ammonia, and the reaction was performed at 35 °C for 6 h. After this was finished, the final product was centrifuged and washed with alcohol twice and deionized water once. The obtained powder was washed with 100 mL of 6% concentrated hydrochloric-methanol solution, and stirred at 60 °C for 6 h. Next, the above mixed solution was washed and centrifuged with deionized water twice, and then the MSNS-NH_2_ was obtained after vacuum drying at 55 °C. Second, as shown in the first step in Figure 1A, 0.313 g of 4-ATP was evenly dispersed in 1 mL PBS, and then 10 mg MSNs-NH_2_ was added to the above solution, which was dissolved by ultrasound. After reacting for 6 h at 37 °C, 4-ATP@MSNs-NH_2_ was obtained.

### 2.3. Synthesis of 4-ATP@MSNs-NH_2_@aptamers

As shown in the second step of Figure 1A, the 4-ATP@MSNs-NH_2_ powder obtained above was dispersed in PBS and sonicated for 2 min to make the 4-ATP@MSNs-NH_2_ more evenly dispersed. Then, 0.5 µM aptamers was added to the above suspension and stirred at 37 °C for 3 h to allow for the full binding of the aptamer to 4-ATP@MSNs-NH_2_. Subsequently, the above solution was centrifuged at 6000 rpm for 5 min to prepare 4-ATP@MSNs-NH_2_@aptamers, and then the unbound 4-ATP and aptamers were removed by washing with dichloromethane and pure water, respectively, and finally re-suspended in PBS and placed at 4 °C for later use.

### 2.4. Synthesis of Ag@MSNs

First, 0.1 g MSNs-NH_2_ powder was dispersed in 20 mL ethanol with constant stirring, then 100 μL MPTMS was added and the reaction was performed at 25 °C for 12 h. After centrifugation at 6000 rpm for 5 min, the powder was washed with pure water and ethanol, respectively, and dried (55 °C, 6 h) for next use. The sulfhydrylated MSNs were sonicated and dispersed in 5 mL water, followed by adding 500 µL 0.1 mol/L AgNO_3_ and reacting at 50 °C for 24 h to obtain Ag@MSNs (Figure 1B).

### 2.5. Feasibility Analysis

4-ATP@MSNs-NH_2_ and 4-ATP@MSNs-NH_2_@aptamers were suspended in PBS, and the supernatant was taken after 15 min and used as blank controls. In addition, 4-ATP@MSNs-NH_2_@aptamers (500 µL) was mixed with the chlorpyrifos standard solution (200 ng/mL, 500 µL) and incubated for 15 min, and the supernatant was collected by centrifugation at 6000 rpm for 5 min. Finally, the supernatant was mixed with the prepared Raman substrate Ag@MSNs in equal volume to measure the Raman signal (Figure 1C).

### 2.6. Sensing Analysis of Chlorpyrifos

The 0.5 µM aptamers were reacted with 4-ATP@MSNs-NH_2_ for 3 h to block the pores. After centrifugation at 6000 rpm for 5 min, the precipitate was taken, and chlorpyrifos of different concentrations (25, 50, 100, 150, 200, 250 ng/mL) was added. After shock incubation for 15 min, the supernatant was obtained by centrifugation at 6000 rpm for 5 min. A mixture of 10 µL of Raman substrate Ag@MSNs and test solution (1:1) was placed on a glass slide, and the SERS spectrum was measured under an incident power of 360.0 mW and laser excitation at 785 nm. The blank control was the absence of chlorpyrifos. All experiments were performed in triplicate. In order to analyze the specificity of the sensor, acetamiprid, carbendazim, and cyhalothrin, which were co-applied with chlorpyrifos in practice or similar to chlorpyrifos in structure, were used for the Raman spectroscopy test with the sensor at a concentration of 200 ng/mL. To determine the reproducibility of the method, six chlorpyrifos solutions were reacted with 4-ATP@MSNs-NH_2_@aptamers and their Raman strengths were measured separately. In addition, the stability of the Raman probe was crucial to evaluate the performance of the method. The Raman probe was stored for 16 days at 4 °C, and the Raman intensity of the Raman probe after reaction with chlorpyrifos was measured every two days. The Raman intensity was compared with the original Raman value to analyze the stability of the Raman probe.

### 2.7. Pretreatment of Actual Samples

Respectively, 100 g of apple and tomato samples were weighed, and then the chlorpyrifos standard was added to the blank samples (0, 15, 30, 60 µg/kg). Subsequently, the above apple and tomato samples were fully ground with a mortar, respectively, and 2 mL 99.5% acetone was added to stand for 5 min. The mixture was centrifuged at 6000 rpm for 5 min, then the supernatant was taken and the volume was fixed to 30 mL with sub-boiling water. The above apple and tomato solutions were filtered through a 0.22 µm filter membrane and the recovery rates of chlorpyrifos were determined by the established sensor, respectively. All experiments were repeated five times.

## 3. Results and Discussion

### 3.1. Characterization of MSNs-NH_2_ and 4-ATP@MSNs-NH_2_

**Figure 2:**
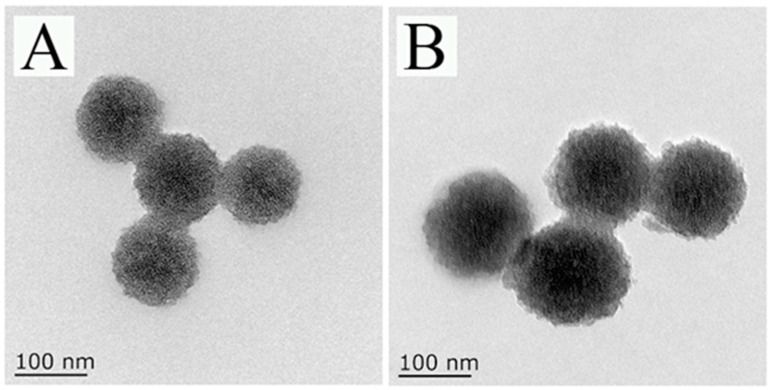
The TEM images of MSNs-NH_2_ (**A**) and 4-ATP@MSNs-NH_2_ (**B**).

TEM was used to characterize the appearance of MSNs-NH_2_ and 4-ATP@MSNs-NH_2_. As shown in Figure 2A, the prepared MSNs-NH_2_ had good dispersion, obvious mesoporous structure, and a uniform particle size with an average size of 115.39 nm. When 4-ATP was loaded, the color of MSNs-NH_2_ changed and the average particle size increased to 146.69 nm (Figure 2B).

**Figure 3:**
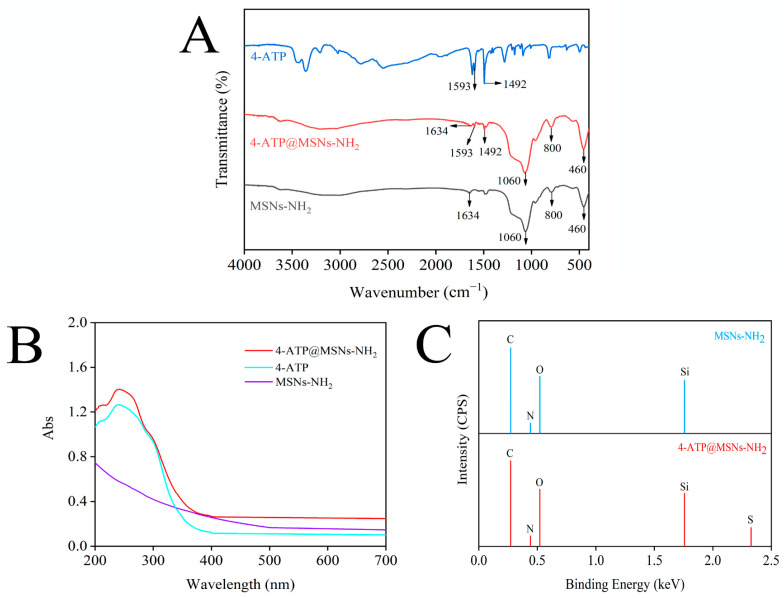
The FTIR (**A**) and UV–Vis absorption spectrum (**B**) of MSNs-NH_2_, 4-ATP, and 4-ATP@MSNs-NH_2_; the EDX spectrum of MSNs-NH_2_ and 4-ATP@MSNs-NH_2_ (**C**).

MSNs-NH_2_, 4-ATP and 4-ATP@MSNs-NH_2_ were further characterized by FTIR. As shown in Figure 3A, MSNs-NH_2_ (black line) exhibited four characteristic peaks at 460 cm^−1^, 800 cm^−1^, 1060 cm^−1^, and 1634 cm^−1^, among which 1060 cm^−1^ and 800 cm^−1^ corresponded to the asymmetric and symmetric tensile vibration peaks of Si–O–Si, 460 cm^−1^ corresponded to O–Si–O bending, and 1634 cm^−1^ corresponded to –NH_2_ bending vibration, indicating successful modification of the amino groups on MSNs. When MSNs-NH_2_ was loaded with 4-ATP (red line), the characteristic peaks of MSNs-NH_2_ were retained, and the same peaks as that of 4-ATP (blue line) appeared at 1492 cm^−1^ and 1593 cm^−1^, which belonged to the aroma-C=C-in-plane vibration, confirming the existence of a benzene ring on MSNs-NH_2_ [30,31]. Meanwhile, the UV absorption spectrum (Figure 3B) shows that 4-ATP has a main absorption peak at 238 nm, and when 4-ATP was combined with MSNs-NH_2_, the peak of 238 nm was still retained. To verify the successful preparation of 4-ATP@MSNs-NH_2_, the elements of MSNs-NH_2_ and 4-ATP@MSNs-NH_2_ were determined by EDX (Figure 3C). MSNs-NH_2_ is mainly composed of C, N, O, and Si elements, while 4-ATP@MSNs-NH_2_ contains S in addition to C, N, O, and Si elements, which is due to the fact that 4-ATP is an aromatic thiol and contains S elements, indicating the successful preparation of 4-ATP@MSNs-NH_2_.

### 3.2. Characterization of 4-ATP@MSNs-NH_2_@aptamers

**Figure 4:**
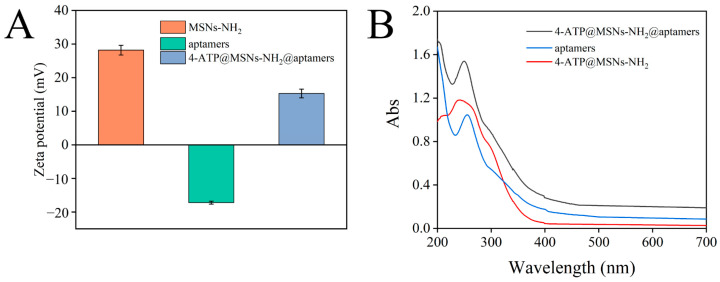
The zeta potential of MSNs-NH_2_, aptamers and 4-ATP@MSNs-NH_2_@aptamers (**A**); the UV−Vis absorption spectrum of ATP@MSNs-NH_2_@aptamers, aptamers, and 4-ATP@MSNs-NH_2_ (**B**).

After binding to 4-ATP@MSNs-NH_2_ through electrostatic interaction, aptamers can play a gating role to control the release of 4-ATP. The binding of aptamer to 4-ATP@MSNs-NH_2_ was verified by the zeta potential and UV absorption spectra. Figure 4A shows that the zeta potential of MSNs-NH_2_ and the aptamers were 28.2 mV and −17.2 mV, respectively. The presence of amino groups on the surface of MSNS results in the positive potential of MSNS-NH_2_. The negative potential of aptamers is due to the negative charge of the carried phosphate. After the aptamers bind to 4-ATP@MSNs-NH_2_, the zeta potential is reduced to 15.3 mV due to the negatively charged aptamers coating the surface of MSNs-NH_2_, which reduces the potential. To confirm the successful binding of the aptamer to 4-ATP@MSNs-NH_2_, the UV absorption spectra were also measured. As shown in Figure 4B, the aptamer had a characteristic absorption peak at 256 nm, and after the aptamers were combined with 4-ATP@MSNs-NH_2_, there was still a characteristic absorption peak at 256 nm, indicating that the aptamers were successfully bound to 4-ATP@MSNs-NH_2_.

### 3.3. Characterization and Activity Analysis of Ag@MSNs

**Figure 5:**
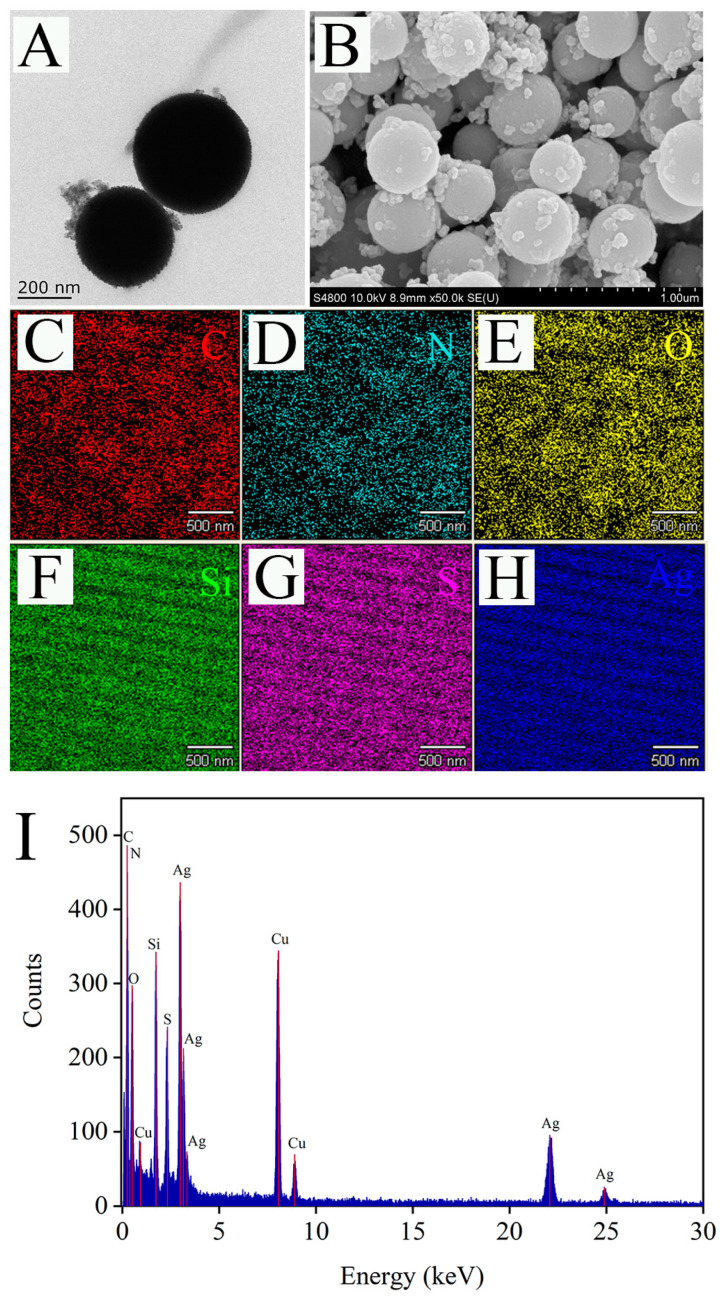
The TEM (**A**), SEM (**B**), mapping images (**C**–**H**), and EDX spectrum (**I**) of Ag@MSNs.

The morphology of the Raman substrate Ag@MSNs was characterized by TEM and SEM. As shown in TEM image, the synthesized Ag@MSNs had a uniform structure and good dispersion (Figure 5A). Similarly, the SEM image showed that Ag@MSNs was spherical and had a rough surface with many irregular protrusions, which were Ag nanoparticles (Figure 5B). Mapping element scanning was used to determine the elemental composition of the Ag@MSNs nanoparticles (Figure 5C–H). The results showed that Ag@MSNs contained C, N, O, Si, S, and Ag elements, which proved the successful synthesis of Ag@MSNs. The EDX spectrum measurement was performed on Ag@MSNs, and elements C, N, O, Si, S, Ag, and Cu (Cu was due to the use of carbon film scaffold during the experiment) were detected, which was consistent with the results of the mapping element scanning (Figure 5I).

**Figure 6:**
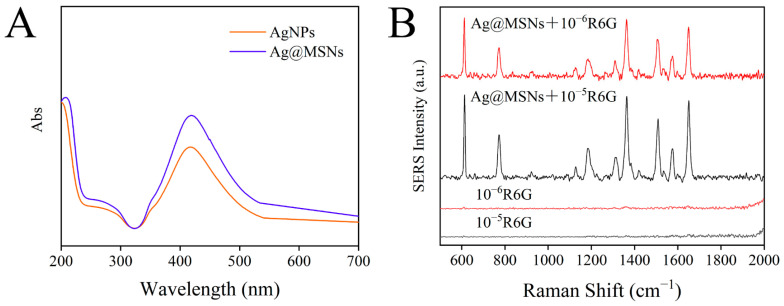
The UV–Vis absorption spectrum of AgNPs and Ag@MSNs (**A**); SERS spectrum of R6G and R6G adsorbed on Ag@MSNs (**B**).

Meanwhile, UV–Vis absorption spectra were used to identify AgNPs and Ag@MSNs. As shown in Figure 6A, AgNPs had an absorption peak at 417 nm, and when AgNPs were combined with MSNs, the absorption peak at 417 nm did not shift, indicating that aggregation did not occur after Ag@MSNs synthesis and the dispersion was good. In addition, a simple experiment was conducted to verify the Raman enhancement ability of the Raman substrate Ag@MSNs. R6G standard solutions (10^−5^ mol/L and 10^−6^ mol/L) were mixed with Ag@MSNs solution at 1:1. Then, 10 µL of the mixture was placed on a glass slide and dried for Raman detection. As shown in Figure 6B, R6G (10^−5^ mol/L and 10^−6^ mol/L) alone had no obvious Raman signal, but the Raman signal was significantly improved after the addition of Ag@MSNs, and the Raman signal was enhanced with the increase in the R6G concentration, which indicated that Ag@MSNs can be used as a highly active Raman substrate for SERS detection.

### 3.4. Feasibility Analysis

**Figure 7:**
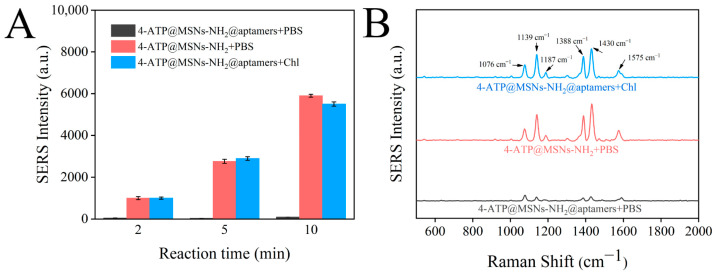
Changes in the SERS intensities of 4-ATP@MSNs-NH_2_, 4-ATP@MSNs-NH_2_@aptamers, and 4-ATP@MSNs-NH_2_@aptamers + chlorpyrifos with time (**A**); SERS spectrum of 4-ATP@MSNs-NH_2_, 4-ATP@MSNs-NH_2_@aptamers, and 4-ATP@MSNs-NH_2_@aptamers + chlorpyrifos (**B**).

The SERS intensities of 4-ATP@MSNs-NH_2_, 4-ATP@MSNs-NH_2_@aptamers, and 4-ATP@MSNs-NH_2_@aptamers + chlorpyrifos were measured using Ag@MSNs as the Raman substrate to verify the feasibility of the sensor. As shown in Figure 7A, with the increase in the incubation time, the Raman signal in the supernatant of 4-ATP@MSNs-NH_2_ without aptamer blocking was gradually enhanced. However, after the addition of aptamers, the Raman signal was low and did not change significantly with the extension of time because 4-ATP could not be released into the supernatant after being blocked by aptamers. After the addition of chlorpyrifos, 4-ATP was released due to the specific binding of the aptamer and chlorpyrifos, and the Raman signal was significantly enhanced, thus ensuring that the sensor is feasible for the detection of chlorpyrifos. Figure 7B shows the Raman spectra after 10 min of reaction, in which the peaks at 1076 cm^−1^, 1187 cm^−1^, and 1575 cm^−1^ were attributed to VC-C + VC-S, βC-H, and VC-C. The peaks at 1139 cm^−1^, 1388 cm^−1^, and 1430 cm^−1^ were attributed to βc-H + VC-N, VN-N + VC-N, and VN-N + βC-H of 4-ATP. Finally, the strongest peak at 1430 cm^−1^ was selected and used for Raman detection.

### 3.5. Optimization of Test Conditions

**Figure 8:**
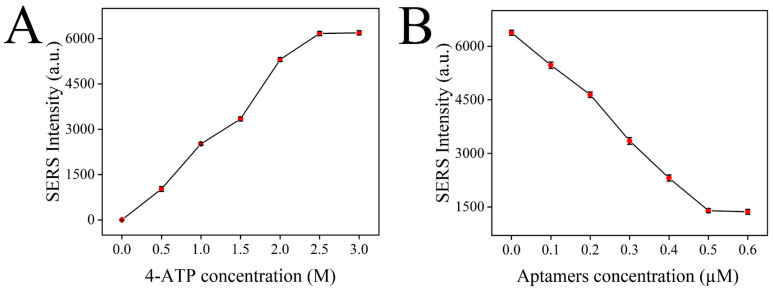
Optimization of the detection conditions: 4-ATP concentration (**A**); aptamer concentration (**B**).

In this detection system, the Raman signal mainly comes from the signal molecule 4-ATP, and the higher the content of 4-ATP in the detection solution, the higher the Raman intensity. Therefore, the content of 4-ATP has an important influence on the sensitivity of the sensor. First, the SERS intensity of the constructed sensor containing different amounts of 4-ATP (0, 0.5, 1, 1.5, 2, 2.5, 3 M) was detected to optimize the content of 4-ATP. As shown in Figure 8A, the SERS intensity of the constructed sensor was continuously enhanced with the increase in the 4-ATP concentration. The release of 4-ATP in the system reached saturation when the concentration of 4-ATP reached 2.5 M, and the SERS signal was no longer enhanced. Therefore, 4-ATP at a concentration of 2.5 M was selected as the optimal concentration for sensor construction.

Aptamer can block the 4-ATP loaded in MSNs, making the release of 4-ATP a controllable process. In the presence of chlorpyrifos, the aptamer on the 4-ATP@MSNs-NH_2_ preferentially binds to chlorpyrifos due to specific adsorption and releases 4-ATP, causing changes in the Raman signal intensity. Therefore, the content of the aptamer is important for the accurate detection of chlorpyrifos. If the content of the aptamer is too low, the blocking effect is not good, which makes the detection result of the sensor inaccurate. If the content of the aptamer is too high, the excess aptamer will be wasted, which greatly increases the detection cost. Therefore, the content of the aptamer should be optimized. In Figure 8B, with the increase in the aptamer concentration, the SERS intensity decreased rapidly. Then, the SERS intensity reached stability when the concentration of the aptamer reached 0.5 µM, so 0.5 µM was selected as the optimal aptamer concentration.

### 3.6. Preparation of SERS Sensor

**Figure 9:**
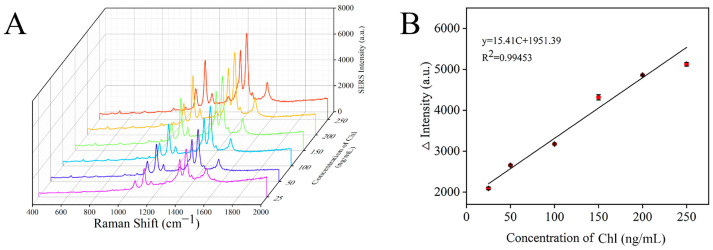
SERS spectra of different concentrations of chlorpyrifos (**A**) and the correlation between Raman intensity and chlorpyrifos concentration (**B**).

Under the optimal conditions, the Raman spectra of chlorpyrifos with different concentrations were detected by a SERS sensor. As shown in Figure 9A, the characteristic peak intensity of the signal molecule 4-ATP in the detection system gradually increased with the increase in chlorpyrifos concentration. At the same time, the relationship curve between the Raman intensity difference at 1430 cm^−1^ and chlorpyrifos concentration was established (Figure 9B). It can be seen that there was a good linear correlation between the Raman intensity and the concentration of chlorpyrifos at 25–250 ng/mL. The linear regression equation was ∆Intensity = 15.41C + 1951.39 (C was the concentration of chlorpyrifos), and the correlation coefficient (R^2^) was 0.99453. ∆Intensity = I − I0, where I and I0 represent the Raman signal intensity at 1430 cm^−1^ in the presence and absence of chlorpyrifos, respectively. Limit of detection (LOD) = 3S/M, where S is the standard deviation of the blank sample, M is the slope of the standard curve, and the calculated LOD was 19.87 ng/mL.

The method constructed in this study was compared with some other methods for chlorpyrifos detection (Table 1). It can be seen that the aptamer SERS sensor constructed in this work has many advantages such as simple sample pretreatment, a convenient and rapid detection process, wide detection linear range, and low LOD, which are suitable for the rapid quantitative detection of chlorpyrifos.

**Table 1:** A comparison of the different methods for the detection of chlorpyrifos.

Method	Linear Range (ng/mL)	LOD (ng/mL)
Bioenzyme sensor [3]	0–100	29.42
Gas chromatography-mass spectrometry [10]	100–1000	100
High-performance liquid chromatography [11]	800–80,000	890
immunochromatographic assay [32]	−	10
dual-readout immunochromatographic assay [33]	0.1–50	0.033
cytometric bead array method [34]	5.14–49.53	1.09
Fluorescence immunoassay [35]	9.77–1250	4.9
Surface-enhanced Raman [36]	−	175.29
This work	25–250	19.87

### 3.7. Specificity, Reproducibility, and Stability of the Sensor

**Figure 10:**
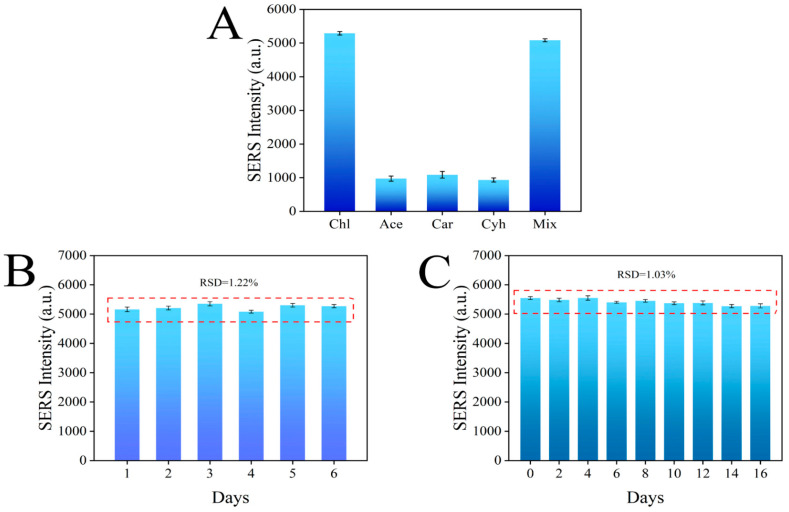
Specificity (**A**), reproducibility (**B**), and stability (**C**) of the aptamer sensor for chlorpyrifos detection.

The specificity test results are shown in Figure 10A, where the Raman signal of chlorpyrifos was much higher than that of the other three pesticides, which was due to the specific binding of the aptamer in the sensor to chlorpyrifos. In addition, the Raman signal intensity of the mixed pesticide was not significantly different from that of chlorpyrifos alone, indicating that other pesticides did not interfere with the sensing system, further proving that the sensor has good selectivity for chlorpyrifos (Figure 10A). The reproducibility of the sensor is very important for the subsequent application. Therefore, six parallel experiments were carried out under the same conditions, and the results showed that the RSD was 1.22%, which proved that the aptamer sensor had good reproducibility (Figure 10B). Finally, the stability of the sensor was tested, and the signal value of the constructed aptamer sensor decreased by less than 2% within 16 days, which proves that the aptamer sensor has good stability (Figure 10C).

### 3.8. Actual Sample Testing

In order to verify the practicability of the aptamer sensor, spiked recovery experiments were carried out on the apple and tomato samples. The recovery rates of chlorpyrifos at three concentration levels in apple and tomato were 90.08–102.2%, with a RSD ranging from 0.64 to 3.32% (Table 2), indicating that the aptamer sensor had good application potential.

## 4. Conclusions

In this study, we developed a simple, rapid, and convenient aptamer-based SERS sensor for the quantitative analysis of chlorpyrifos residues in food samples. Under the optimal experimental conditions, there was a good linear correlation between the Raman intensity and the concentration of chlorpyrifos at 25–250 ng/mL. The linear regression equation was ∆Intensity = 15.41C + 1951.39 (C was the concentration of chlorpyrifos), the correlation coefficient R^2^ = 0.99453, and the LOD was 19.87 ng/mL. The recovery rate of chlorpyrifos in apple and tomato were 90.08–102.2%, with a RSD lower than 3.32%, indicating that this method has good practical application value. In general, the aptamer sensor constructed in this study has a simple pretreatment and operation process, wide linear range, high sensitivity, good specificity, reproducibility and stability, and low cost. Therefore, this study has significant guidance and reference for the detection of chlorpyrifos in the environment and agricultural products. In addition, the aptamer sensor constructed in this study is also suitable for the detection of other types of pesticides and compounds, but the conditions need to be re-optimized according to the specific situation in order to achieve better sensitivity and accuracy.

## Figures and Tables

**Table 2:** Determination of chlorpyrifos in the actual samples.

Sample	Spiked Amount (µg/kg)	Measured Amount (ng/mL)	Recovery Rate (%)	RSD (%)
Apple	0	ND	ND	ND
	15	51.1	102.2	1.24
	30	90.08	90.08	1.71
	60	198.26	99.13	1.11
Tomato	0	ND	ND	ND
	15	50.31	100.63	0.64
	30	99.42	99.42	3.32
	60	200.15	100.07	1.71

## Data Availability

The data presented in this study are available on request from the corresponding author.
